# Early Life Intervention Using Probiotic *Clostridium butyricum* Improves Intestinal Development, Immune Response, and Gut Microbiota in Large Yellow Croaker (*Larimichthys crocea*) Larvae

**DOI:** 10.3389/fimmu.2021.640767

**Published:** 2021-03-08

**Authors:** Zhaoyang Yin, Qiangde Liu, Yongtao Liu, Shengnan Gao, Yuliang He, Chuanwei Yao, Wenxing Huang, Ye Gong, Kangsen Mai, Qinghui Ai

**Affiliations:** ^1^ Key Laboratory of Aquaculture Nutrition and Feed (Ministry of Agriculture and Rural Affairs), Key Laboratory of Mariculture (Ministry of Education), Ocean University of China, Qingdao, China; ^2^ Laboratory for Marine Fisheries Science and Food Production Processes, Qingdao National Laboratory for Marine Science and Technology, Qingdao, China

**Keywords:** *Clostridium butyricum*, marine fish larvae, early life intervention, intestinal development, immune response, gut microbiota

## Abstract

Marine fish larvae are vulnerable during the early life period. The early intervention using probiotics may be a promising method to improve growth of fish larvae. In this study, a 30-day feeding trial was conducted to evaluate the effects of early life intervention using probiotic *Clostridium butyricum* (CB) on growth performance, intestinal development, immune response and gut microbiota of large yellow croaker (*Larimichthys crocea*) larvae. Four isonitrogenous and isolipidic diets were formulated with the supplementation of four different levels of CB (5 × 10^9^ CFU g^−1^), 0.00% (Control), 0.10% (CB1), 0.20% (CB2), and 0.40% (CB3). Results showed that larvae fed diets with CB had significant higher final length than the control group. Meanwhile, larvae fed the diet with 0.10% CB had significant higher final weight and specific growth rate (SGR) than the control group. However, no significant difference in survival rate was observed among dietary treatments. CB supplementation significantly increased the height of intestinal villus and the length of intestinal enterocyte. Similarly, CB supplementation significantly increased the expression of tight zonula occludens-2 (*zo-2*) and ornithine decarboxylase (*odc*) than the control group. Larvae fed the diet with 0.20% CB had significant higher lipase and leucine-aminopeptidase (LAP) activity than the control group. Moreover, CB supplementation significantly improved immune enzyme activities than the control group. Sequencing of bacterial 16S rRNA V4-5 region indicated that dietary CB altered intestinal microbiota profile and decreased intestinal microbial diversities of larvae. CB supplementation could effectively increase the abundance of CB, and decrease the abundance of some potential pathogenic bacteria in larval gut. These results revealed that early life intervention using 0.10–0.20% CB could promote growth of large yellow croaker larvae probably through promoting intestinal development, improving immune enzyme activities and modulating gut microbiota.

## Introduction

Marine fish gut tract is not fully developed during the early stage of life (1). Fish larvae undergo major morphological, cellular and functional changes of gut tract during the first stage of life (2). The successful development of gut tract is important for fish larvae to digest and absorb food (3). Furthermore, gut microbiota is not fully assembled in marine fish digestive tract during the early stage of life (4 **).** The microbes that reside in the gut tract have profound influence on host immune system priming, protection and development, as well as nutrient supplementation to the host (5, 6). The rate and trajectory of acquisition of gut microbiota have a considerable impact on later health outcomes (7).

In recent years, many studies have indicated that probiotic treatment in the early life is necessary for digestive physiology functions, gastrointestinal tract immune and microbiota development (8, 9). *Clostridium butyricum* (CB) is a gram-positive, obligate anaerobic and endospore-forming probiotic, which is part of the normal gut bacteria for both mammals and aquatic animals (10, 11). CB could improve gastrointestinal function, which has been used for the clinical treatment of newborn animals (12–14). In recent years, research studies in regard to CB were carried out in aquatic species, including tilapia (15), shrimp (16), and prawn (17). However, to our best knowledge, the role of dietary CB in marine fish larvae is still uncertain.

Large yellow croaker (*Larimichthys crocea*) is an economic important marine fish species in south China (18). Similarly to most marine fish species, large yellow croaker larvae had an undifferentiated and undeveloped gut and accessory organs (19). The production of large yellow croaker is often hampered by high mortality in larval stage (20). Therefore, the present study was conducted to investigate the effects of early life intervention using CB on the growth performance, immune response, intestinal development and gut microbiota of large yellow croaker (*Larimichthys crocea*) larvae. By using a marine fish larvae model, the present study might provide novel insights into probiotic treatment in early life intervention.

## Materials and Methods

### Animal Ethics

All procedures of this study were conducted on fish, and animal care was performed strictly followed the Management Rule of Laboratory Animals (Chinese Order No. 676 of the State Council, revised 1 March 2017).

### Diet Formulation

Large yellow croaker larvae were fed with four isonitrogenous (52% crude protein) and isolipidic (19% crude lipid) diets, which were supplemented with different levels of CB (0.00% as the control group, 0.10% as CB1 group, 0.20% as CB2 group, and 0.40% as CB3 group) (**Supplementary Table 1**). The additive CB was purchased from Vland Biotech Co., Ltd., China, containing cells at 5 × 10^9^ colony forming units (CFU) g^−1^. Micro-diet (MD) was manufactured by micro-bonding technology (20). The particle size of the formulated diets ranged from 250 to 380 μm for fish larvae between 14 and 24 days after hatch (DAH) and 380–500 μm for fish larvae between 25 and 47 DAH.

### Experimental Procedure

The objective animal of this study were purchased from the Ningde Fufa Fishery Company Limited, Ningde, China, and were reared at the State Key Laboratory of Large Yellow Croaker, Ningde, China. All fish larvae in the hatchery were fed with rotifers, *Brachionus plicatilis* (0.5–2.0 × 10^4^ individual L^−1^) from 3 to 7 DAH, *Artemia nauplii* (1.0–1.5 × 10^3^ individual L^−1^) from 6 to 11 DAH, and *Calanus sinicus* from 10 to 14 DAH, and then the larvae were weaned onto experimental diets. Ten thousand larvae (17 DAH) were randomly allocated in 12 blue plastic tanks (water volume 1,000 L) to conduct the experiment. Different experimental diets were randomly allocated to triplicate groups of larvae. During the rearing period, water temperature, pH, and salinity were strictly controlled during the rearing period, ranging from 22 to 24°C, 7.9 to 8.3, 21 to 24‰, respectively. The water volume was renewed about 60–150% daily. Larvae were reared under 16h light:8h dark dial cycle photoperiod. All the larvae were manually fed to satiation with the experimental diets seven times daily (6:00, 8:00, 11:00, 14:00, 17:00, 20:00, and 22:00) during the whole experiment period (from 17 DAH to 47 DAH).

### Sample and Dissection

Before the experiment was conducted, initial body weight and body length of 150 randomly collected larvae (17 DAH) were measured. At the end of the experiment, larvae were fasted for 24 h before sampling. Survival rate was calculated by comparing the remaining larvae in each tank to the initial number. Final body weight and final body length of 1,000 larvae randomly collected from each tank were measured. Thirty larvae were collected from each tank and preserved in 4% paraformaldehyde for 24 h and transferred to 75% alcohol for hematoxylin and eosin (H&E) staining. Ninety larvae from each tank were dissected on ice to obtain visceral mass, which contains a crude mixture of pancreas, liver, heart, spleen and intestine, for immune enzyme activity assay. Pancreatic and intestinal segments (PS and IS) of sixty larvae were separated on a glass plate maintained at 0°C under a dissecting microscope for digestive enzyme activity assay (20). The whole intestine of sixty larvae from each tank was separated aseptically under a dissecting microscope for the analysis of intestinal microflora and gene expression assays.

### Analytical Methods

#### Digestive Enzyme Activities Assay

Four stages (22, 27, 37, and 47 DAH) of large yellow croaker larvae were chosen to reflect digestive enzyme activities during different growth stages (1). PS and IS (0.1–0.2 g) of 22, 27, 37, and 47 DAH larvae were weighed and homogenized in 2 ml 0°C normal saline, followed by centrifuging (2,500g, 10 min), then the supernatant was collected for further assay. Purified brush border membranes (BBMs) from homogenate of the intestinal segment were obtained according to a method described by published paper (21). Activities of leucine-aminopeptidase (LAP) were assayed in BBM according to published paper (22). Trypsin activity was assayed following published paper (23). Amylase activity was assayed with *α*-Amylase Assay Kit (Nanjing Jiancheng Bio-Engineering Institute, China). Lipase activity was assayed with Lipase Assay Kit (Nanjing Jiancheng Bio-Engineering Institute, China). Protein was determined by the Total protein quantitative assay kit (Nanjing Jiancheng Bio-Engineering Institute, China).

#### Immune Enzyme Activities Assay

The visceral mass was weighed and homogenized in phosphate-buffered saline. The proportion of tissue (g) and saline (ml) was 1:9. The homogenate of larvae was then centrifuged (4,000g, 15 min), and the supernatant was collected for the following assays. Immune enzymes kits (Nanjing Jiancheng Bio-Engineering Institute, China) were used to evaluate the activity of acid phosphatase (ACP), alkaline phosphatase (AKP), catalase (CAT), lysozyme (LZM), and superoxide dismutase (SOD) in the collected visceral mass.

#### Intestinal Histology Analysis

The intestinal micromorphology was determined based on the method described by published paper (19). Briefly, the whole larvae were washed and dehydrated with gradient alcohol, and then paraffin-embedded, sectioned, and stained with hematoxylin and eosin.

#### cDNA Synthesis and Real-Time Quantitative Polymerase Chain Reaction

Total RNA was extracted from the whole intestine of larvae using RNAiso Plus (Takara Biotech, Dalian, China) following the instructions of the manufacturer, and electrophoresed on a 1.2% denaturing agarose gel to detect the quality (20). And then extracted RNA was assessed by a Nano Drop^®^2000 spectrophotometer (Thermo Fisher Scientific, USA) to test the concentration. Then, RNA reverse was transcribed to cDNA by Prime Script-RT reagent Kit (Takara, Japan). The real-time quantitative polymerase chain reaction was carried out in a quantitative thermal cycler (CFX96TM Real-Time System, BIO-RAD, USA). The operational approach referred to Zuo et al. (24). The primer sequences for proliferating cell nuclear antigen (*pcna*), tight zonula occludens-1 (*zo-1*), tight zonula occludens-2 (*zo-2*), *occludin*, ornithine decarboxylase (*odc*), interleukin-1*β* (*il-1β*), interleukin-6 (*il-6*), interleukin-8 (*il-8*), interferon *γ* (*ifnγ*), cyclooxygenase-2 (*cox-2*) and *β-actin* were designed and synthesized based on the corresponding sequences in GenBank and published papers (**Supplementary Table 2**) (20, 25, 26). The fluorescence data acquired during the extension phase were normalized to *β-actin via* 2^−ΔΔCT^ methods (27).

#### Gut Microbiota Collection and Bacterial Genomic DNA Extraction

Thirty fresh larvae gut of 47 DAH were mixed and stored at −80°C until DNA was extracted using the CTAB method (28). 1 ng of purified gut bacterial DNA was used to sequence and classify as previously described (29). The 515f/907r primer set amplified V4-5 region of the 16s rRNA gene was used to conduct PCR, using barcoded primers: Fwd5′-GTGCCAGCMGCCGCGGTAA-3′, Rev5′-CCGTCAATTCCTTTGAGTTT-3′. Subsequently, sequencing was performed on an Illumina MiSeq platform, provided by Beijing Novogene Genomics Technology Co. Ltd. (China). Complete data were submitted to the NCBI Sequence Read Archive (SRA) database under accession number PRJNA686247. FLASH (V1.2.7, http://ccb.jhu.edu/software/FLASH/) was used to merge the reads from the same original DNA (30). Uparse software (Uparse v7.0.1001, http://drive5.com/uparse/) was applied to cluster the unique sequences to acquire operational taxonomic units (OTUs) according to the similarity of sequence distance based up to 97% or greater (31). Subsequently, the representative OTUs were annotated through the RDP Classifier (Version 2.2, http://sourceforge.net/projects/rdp-classifier/) and GreenGene database (http://greengenes.lbl.gov/cgi-bin/nph-index.cgi) (32). QIIME (Quantitative Insights Into Microbial Ecology) V 1.7.0 software package (http://qiime.org/index.html) and the UPARSE (http://drive5.com/uparse/) pipeline were adopted to analyze the alpha and beta diversity. Principal component analysis (PCA) was conducted with Mothur and R software packages (http://www.R-project.org). Linear discriminant analysis (LDA) effect size (LefSe) analysis was used to identify the different abundant taxa between the control group and different CB groups (33).

### Calculations and Statistical Analysis

The growth parameters were calculated as follows:

$$Survivalrate(\%)=N_{t}\times 100/N_{i}$$

$$Specificgrowthrate(SGR,\%\;day^{-1})=(LnW_{t}-LnW_{i})\times 100/d$$

Where N_t_ is final number of larvae in each tank and N_i_ is initial number of larvae in each tank at the beginning of the experiment; W_t_ and W_i_ are the final and initial body weights, respectively; d is the experimental period in days.

Statistical analysis was performed in SPSS 16.0 (SPSS Inc., USA). Data from each treatment were subjected to one-way analysis of variance (ANOVA) followed by Tukey’s multiple-range test. For statistically significant differences, *P <*0.05 was applied. Results were presented as mean ± S.E. (Standard error of means).

## Results

### Early Life Intervention With CB Improved Growth of Fish Larvae

No significant difference in survival rate was observed among dietary treatments (*P* > 0.05) (**Table 1**), whereas the highest value was recorded in larvae fed diets with 0.10% CB, followed by 0.20% CB, 0.40% CB and 0.00% CB, respectively. With the increase of CB level in diets, the growth performance firstly increased and then decreased. CB supplementation significantly improved final body length than the control group (*P* < 0.05) (**Table 1**). Meanwhile, larvae fed the diet with 0.10% CB showed significant higher final body weight and SGR than the control group (*P* < 0.05) (**Table 1**).

**Table 1:** Effects of early life intervention using *Clostridium butyricum* on growth performance of large yellow croaker larvae (Means ± S.E., n = 3)^1^.

Parameters	Experimental diets (CB%)			
	Control (0.00%)	CB1 (0.10%)	CB2 (0.20%)	CB3 (0.40%)
Initial weight (mg)	3.67 ± 0.11	3.67 ± 0.11	3.67 ± 0.11	3.67 ± 0.11
Final weight (mg)	72.57 ± 2.12^b^	97.12 ± 2.76^a^	88.68 ± 5.33^ab^	88.53 ± 4.41^ab^
Initial length (mm)	6.43 ± 0.07	6.43 ± 0.07	6.43 ± 0.07	6.43 ± 0.07
Final length (mm)	15.57 ± 0.30^b^	17.48 ± 0.12^a^	17.00 ± 0.21^a^	17.07 ± 0.29^a^
Survival rate (%)	15.26 ± 1.87	23.20 ± 2.60	20.35 ± 1.77	19.83 ± 2.05
SGR (%/day) ^2^	9.95 ± 0.10^b^	10.92 ± 0.09^a^	10.60 ± 0.21^ab^	10.60 ± 0.17^ab^

^1^Data in the same row sharing the same superscript letter are not significantly different as determined by Tukey’s test (P > 0.05).

^2^SGR, Specific growth rate.

### Early Life Intervention With CB Improved Gut Tract Development

#### Early Life Intervention With CB Improved the Intestinal Morphology

CB supplementation effectively improved the intestinal morphology of large yellow croaker larvae (**Supplementary Figure S1**). Larvae fed diets with CB had significant higher villus height and enterocyte height than the control group (*P* < 0.05) (**Table 2**). Similarly, significant higher muscular thickness was observed in fish fed the diet with 0.10% CB than the control group (*P* < 0.05) (**Table 2**).

**Table 2:** Effects of early life intervention using *Clostridium butyricum* on morphology of the intestine of large yellow croaker larvae (Means ± S.E., n = 3)^1^.

Parameters	Diets (CB%)			
	Control (0.00%)	CB1 (0.10%)	CB2 (0.20%)	CB3 (0.40%)
Villus height μm	84.30 ± 13.56^b^	155.29 ± 3.73^a^	151.45 ± 15.81^a^	151.16 ± 11.62^a^
Enterocyte height μm	12.73 ± 1.41^b^	18.74 ± 0.21^a^	18.87 ± 1.70^a^	19.83 ± 1.00^a^
Muscular thickness μm	14.23 ± 3.10^b^	27.03 ± 2.90^a^	20.98 ± 1.83^ab^	21.99 ± 0.42^ab^

^1^Data in the same row sharing the same superscript letter are not significantly different as determined by Tukey’s test (P > 0.05).

#### Early Life Intervention With CB Increased Gene Expression of zo-2 and odc

**Figure 1:**
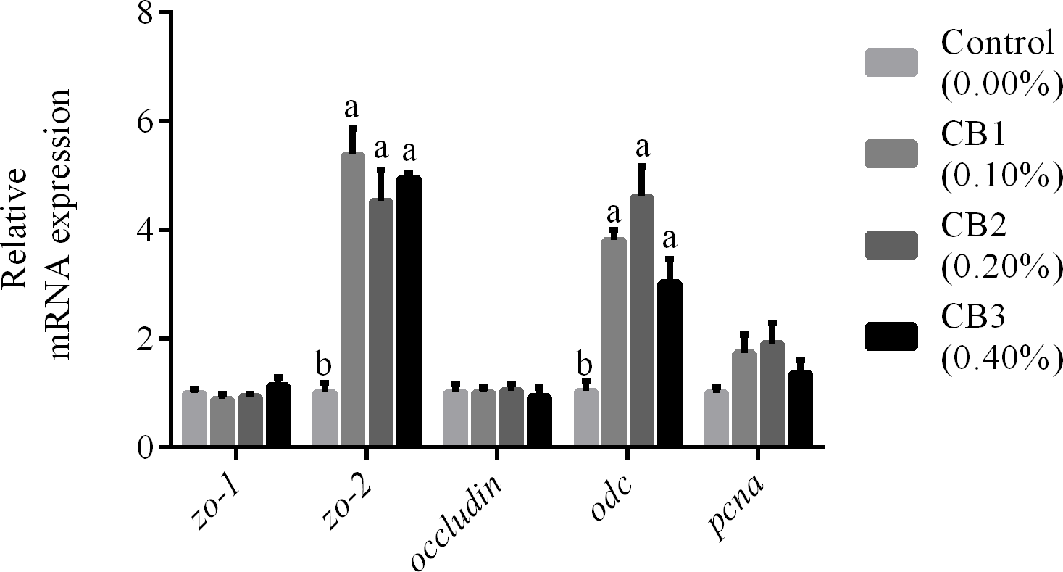
Effects of early life intervention using *Clostridium butyricum* on *zo-1*, *zo-2*, *occludin*, *odc* and *pcna* mRNA expression in whole intestine of large yellow croaker larvae. Values are means (n = 3), with their standard errors represented by vertical bars. Bars bearing the same letters were not significantly different (*P > *0.05, Tukey’s test).

The markers for epithelial proliferation and differentiation were selected (*odc*, *pcna*, *zo-1*, *zo-2* and *occludin*) to compare intestinal development among larvae fed diets with different levels of CB. CB supplementation significantly increased mRNA expression of *zo-2* and *odc* than the control group (*P* < 0.05) (**Figure 1**). However, no significant difference in mRNA expression of *zo-1*, *occludin*, and *pcna* was observed among dietary treatments (*P* > 0.05) (**Figure 1**).

#### Early Life Intervention With CB Improved Lipase and LAP Activities

No significant difference in the activity of trypsin and amylase in IS and PS was observed among dietary treatments (*P* > 0.05) (**Table 3**). Compared with the control group, 0.20% CB supplementation not only increased lipase activity of 37 DAH larvae in PS and IS, but also increased lipase activity of 47 DAH larvae in IS (*P* < 0.05) (**Table 3**). Similarly, 0.20% CB supplementation significantly increased LAP activity of 47 DAH larvae in BBM than the control group (*P* < 0.05) (**Table 3**).

**Table 3:** Effects of early life intervention using *Clostridium butyricum* on digestive enzyme activity of large yellow croaker larvae (Means ± S.E., n = 3)^1^.

Parameters		Date	Experimental diets (CB%)			
			Control (0.00%)	CB1 (0.10%)	CB2 (0.20%)	CB3 (0.40%)
Lipase^2,4^	PS^4^	22 DAH	3.14 ± 0.18	3.16 ± 0.25	3.97 ± 0.35	4.36 ± 0.42
		27 DAH	3.87 ± 0.27	3.76 ± 0.16	4.44 ± 0.49	4.77 ± 0.84
		37 DAH	4.65 ± 0.14^b^	3.94 ± 0.51^b^	7.68 ± 0.13^a^	3.67 ± 0.55^b^
		47 DAH	2.74 ± 0.16	2.54 ± 0.17	2.96 ± 0.11	2.93 ± 0.02
	IS^4^	22 DAH	1.78 ± 0.07	2.22 ± 0.33	2.43 ± 0.23	2.17 ± 0.11
		27 DAH	3.31 ± 0.88	3.49 ± 0.28	3.76 ± 0.25	3.57 ± 0.56
		37 DAH	6.06 ± 0.26^b^	6.32 ± 0.64^b^	8.56 ± 0.25^a^	7.39 ± 0.26^ab^
		47 DAH	2.78 ± 0.20^b^	5.18 ± 0.86^ab^	6.15 ± 0.85^a^	5.42 ± 0.82^ab^
Trypsin^3,4^	PS	22 DAH	0.99 ± 0.02	1.24 ± 0.11	1.16 ± 0.07	1.31 ± 0.05
		27 DAH	2.12 ± 0.18	2.08 ± 0.09	2.19 ± 0.12	2.14 ± 0.20
		37 DAH	1.89 ± 0.20	2.06 ± 0.10	2.22 ± 0.33	2.03 ± 0.41
		47 DAH	2.33 ± 0.32	2.97 ± 0.40	2.25 ± 0.43	2.09 ± 0.07
	IS	22 DAH	3.79 ± 0.87	3.12 ± 0.14	5.34 ± 0.51	4.20 ± 1.08
		27 DAH	1.37 ± 0.07	1.45 ± 0.26	1.19 ± 0.06	1.38 ± 0.18
		37 DAH	1.84 ± 0.21	1.91 ± 0.12	1.91 ± 0.44	1.74 ± 0.15
		47 DAH	6.19 ± 0.63	8.84 ± 0.89	7.20 ± 0.87	5.68 ± 0.13
Amylase^3,4^	PS	22 DAH	0.21 ± 0.01	0.25 ± 0.04	0.29 ± 0.00	0.23 ± 0.02
		27 DAH	0.24 ± 0.02	0.26 ± 0.01	0.27 ± 0.04	0.23 ± 0.03
		37 DAH	0.47 ± 0.01	0.43 ± 0.03	0.42 ± 0.01	0.55 ± 0.07
		47 DAH	0.50 ± 0.02^ab^	0.61 ± 0.03^a^	0.47 ± 0.03^b^	0.60 ± 0.02^a^
	IS	22 DAH	0.49 ± 0.02	0.46 ± 0.01	0.46 ± 0.01	0.50 ± 0.03
		27 DAH	0.37 ± 0.07	0.40 ± 0.06	0.43 ± 0.09	0.41 ± 0.10
		37 DAH	0.42 ± 0.03	0.42 ± 0.03	0.58 ± 0.06	0.57 ± 0.07
		47 DAH	0.47 ± 0.03	0.44 ± 0.05	0.40 ± 0.01	0.37 ± 0.02
LAP^2,4,5^	BBM^4^	22 DAH	7.00 ± 0.90	5.67 ± 0.50	6.55 ± 1.79	7.19 ± 0.36
		27 DAH	3.25 ± 0.47	3.82 ± 0.61	3.17 ± 0.36	4.57 ± 0.87
		37 DAH	19.29 ± 2.29	14.39 ± 1.41	12.67 ± 1.13	11.61 ± 2.93
		47 DAH	43.42 ± 1.76^b^	52.80 ± 2.58^ab^	70.24 ± 6.40^a^	39.71 ± 4.15^b^

^1^Data in the same row sharing the same superscript letter are not significantly different as determined by Tukey’s test (P > 0.05).

^2^The units of enzyme activity is U/g protein.

^3^The unit of enzyme activity is U/mg protein.

^4^PS, pancreatic segments; IS, intestinal segments; BBM, brush border membrane.

^5^LAP, leucine-aminopeptidase.

### Early Life Intervention With CB Improved Immune Response

With the increase of CB level in diets, immune enzyme activities firstly increased and then decreased. CB supplementation significantly improved LZM activity than the control group (*P* < 0.05) (**Table 4**). Furthermore, 0.10 and 0.20% CB supplementation significantly increased AKP and CAT activities than the control group (*P* < 0.05) (**Table 4**). 0.10% CB supplementation significantly increased ACP activity than the control group (*P* < 0.05) (**Table 4**). However, no significant difference in the activity of SOD was observed among dietary treatments (*P* > 0.05) (**Table 4**). Similarly, no significant difference in mRNA expression of inflammatory factors (*il-1β*, *il-6*, *il-8*, *ifnγ* and *cox-2*) was observed among dietary treatments (*P* > 0.05) (**Supplementary Figure 2**).

**Table 4:** Effects of early life intervention using *Clostridium butyricum* on immune enzyme activity in the visceral mass of large yellow croaker larvae (Means ± S.E., n = 3)^1^.

Parameters	Diets (CB%)			
	Control (0.00%)	CB1 (0.10%)	CB2 (0.20%)	CB3 (0.40%)
ACP^2,4^	663.78 ± 69.96^b^	1179.07 ± 133.34^a^	1041.85 ± 65.69^ab^	1054.63 ± 68.31^ab^
AKP^2,4^	1014.21 ± 234.90^b^	2456.50 ± 252.84^a^	2091.23 ± 127.03^a^	1876.39 ± 102.76^ab^
CAT^3,4^	7.62 ± 3.34^b^	26.92 ± 4.71^a^	33.90 ± 2.02^a^	22.01 ± 1.77^ab^
LZM^3,4^	0.60 ± 0.08^c^	1.32 ± 0.10^b^	2.09 ± 0.10^a^	2.12 ± 0.13^a^
SOD^3,4^	112.43 ± 9.06	105.23 ± 8.84	96.06 ± 4.40	105.99 ± 11.22

^1^Data in the same row sharing the same superscript letter are not significantly different as determined by Tukey’s test (P > 0.05).

^2^The unit of enzyme activity is U/g protein.

^3^The unit of enzyme activity is U/mg protein.

^4^ACP, acid phosphatase; AKP, alkaline phosphatase; CAT, catalase; LZM, lysozyme; SOD, superoxide dismutase.

### Early Life Intervention With CB Altered Gut Microbiota of Fish Larvae

#### Structural Changes of Gut Microbiota in Response to CB Supplementation

After assembled, quality screened and trimmed, a total of 768,414 high quality valid reads was obtained, ranging from 56,190 to 69,519, resulting in identification of 1,850 OTUs with 97% identity from 12 samples (data not shown). The OTUs were assigned to 326 species, 547 genera, 240 families, 117 orders, 49 class, and 30 phyla (data not shown). For all samples, the rarefied curves for OTU number tended to approach the saturation plateau, indicating complete sequencing efforts for all samples (**Supplementary Figure 3**).

**Figure 2:**
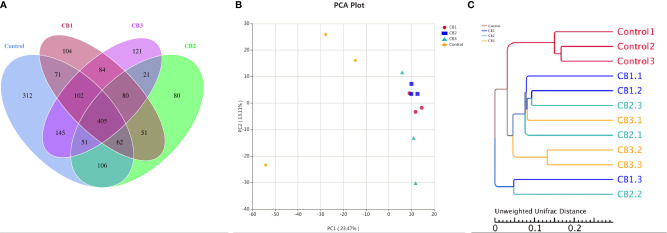
Effects of early life intervention using *Clostridium butyricum* on intestinal microbial structure of large yellow croaker larvae (n = 3/group). **(A)** Venn diagram; **(B)** principal component analysis (PCA); **(C)** unweighted uniFrac distance matrix.

A Venn diagram showed that 106 OTUs were shared by group Control, CB1, CB2 and CB3, and the number of unique OTUs in group Control, CB1, CB2 and CB3 was 312, 104, 80 and 121, respectively (**Figure 2A**). The alpha diversity index results indicated that the inclusion of dietary CB led to lower richness of observed species number in large yellow croaker larvae gut microbiota (*P* < 0.05) (**Table 5**). Alpha diversity including phylogenetic diversity whole tree (PD whole tree) index and abundance-based coverage estimator (ACE) index were markedly decreased in the gut microbiota of larvae in CB groups (*P* < 0.05) (**Table 5**). Meanwhile, larvae fed the diet with 0.20% CB showed significant lower Shannon index in the gut microbiota (*P* < 0.05) (**Table 5**). To analyze the extent of similarities in microbial communities, PCA was conducted to determine the beta diversity (**Figure 2B**). The PCA results showed that the intestine bacterial in the control group and CB groups were separated (**Figure 2B**). Similarly, the gut microbiota structure from groups CB1, CB2, and CB3 were similar and clustered within one higher branch, whereas the control group was distinct from the CB groups and formed another branch (**Figure 2C**).

**Table 5:** Effects of early life intervention using *Clostridium butyricum* on alpha diversity index of intestinal microbiota of large yellow croaker larvae (Means ± S.E., n = 3)^1^.

Parameters	Richness estimates		Diversity estimates		
	Observed species	ACE^2^	Shannon	PD whole tree^3^	Simpson
Control (0.00%)	882 ± 17^a^	993.28 ± 9.59^a^	5.42 ± 0.20^a^	76.49 ± 0.97^a^	0.92 ± 0.01
CB1 (0.10%)	550 ± 36^b^	675.74 ± 45.73^b^	4.73 ± 0.17^ab^	52.31 ± 4.51^b^	0.90 ± 0.01
CB2 (0.20%)	485 ± 23^b^	608.71 ± 31.99^b^	3.88 ± 0.22^b^	46.11 ± 2.84^b^	0.83 ± 0.04
CB3 (0.40%)	580 ± 37^b^	714.65 ± 60.65^b^	4.61 ± 0.42^ab^	59.56 ± 4.11^b^	0.88 ± 0.05

^1^Data in the same column sharing the same superscript letter are not significantly different as determined by Tukey’s test (P > 0.05).

^2^ACE, abundance-based coverage estimator.

^3^PD whole tree: phylogenetic diversity whole tree.

#### Component Changes of Gut Microbiota in Response to CB Supplementation

**Figure 3:**
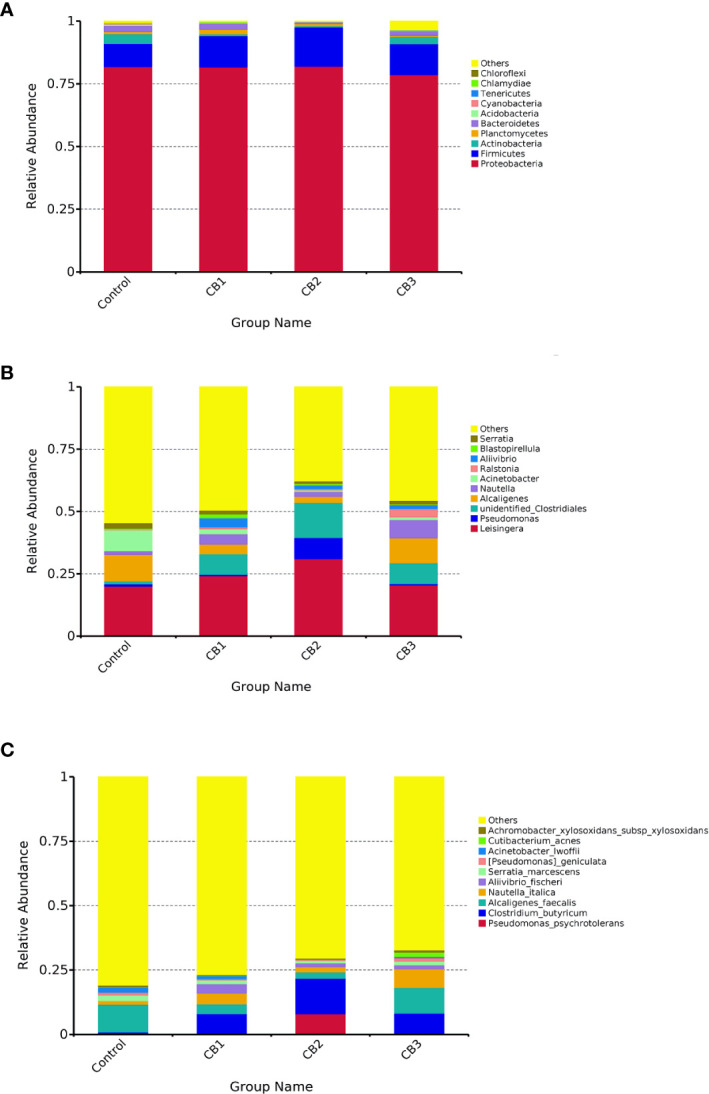
Taxonomy classification of reads at phylum **(A)**, genus **(B)** and specie **(C)** taxonomic levels (n = 3/group). Only top 10 most abundant (based on relative abundance) bacterial phyla, genera and species were shown. Other phyla, genera and species were all assigned as ‘Others’.

At the phylum level, Proteobacteria, Firmicutes and Actinobacteria were detected as predominant bacterial phyla in the intestine of large yellow croaker from all groups (**Figure 3A**). At the genus level, *Leisingera*, *Pseudomonas* and unidentified *Clostridiales* were detected as predominant bacterial genera in the intestine of large yellow croaker larvae from all groups (**Figure 3B**). At the species level, *Pseudomonas psychrotolerans*, *Clostridium butyricum*, *Alcaligenes faecalis* composed dominant species of larval gut microbiota communities (**Figure 3C**).

**Figure 4:**
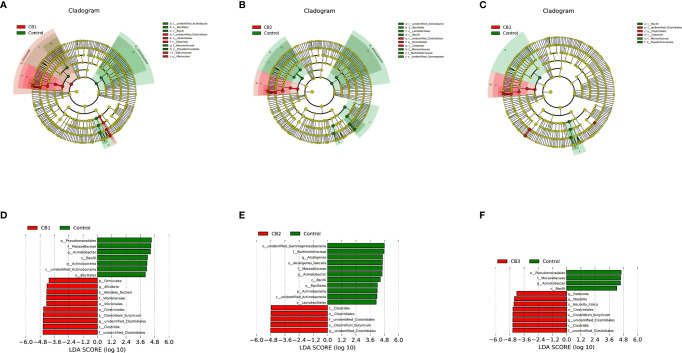
LEfSe analysis identified the most differentially abundant taxons between Control and *Clostridium butyricum* supplementation groups (n = 3/group). **(A–C)** Taxonomic representation of statistically and biologically consistent differences among intestinal microbiota of large yellow croaker fed with Control, CB1, CB2 and CB3 diets. Differences were represented by the color of the most abundant taxa (Green indicated one taxon with significantly higher relative abundance in Control treatment, red indicated one taxon significantly higher in CB treatment and yellow indicated no significant difference). **(D–F)** Histogram of linear discriminant analysis (LDA) scores for differentially abundant taxon. Cladogram was calculated by LefSe, and displayed according to effect size.

To compare the differences in gut microbial community composition between the control group and CB groups, LEfSe analysis was performed (**Figure 4**). The results revealed that there were significant differences in taxonomic distribution of intestinal microbiota communities between the control group and CB groups. CB supplementation significantly increased the relative abundance of class Clostridia, order Closrtidiales, family unidentified Clostridiales, genus unidentified *Clostridiales* and specie *Clostridium butyricum*, while decreased the relative abundance of class Bacilli, family Moraxellaceae and genus *Acinetobacter* (*P* < 0.05) (**Figures 4A–F**). Furthermore, dietary CB at 0.10% level significantly increased the relative abundance of phylum Firmicutes, order Vibrionales, family Vibrionaceae, genus *Aliivibrio* and specie *Aliivibrio fischeri*, but significantly decreased the relative abundance of phylum Actinobacteria, class unidentified Actinobacteria, orders Bacillales and Pseudomonadales (*P* < 0.05) (**Figures 4A, D** **)**. Dietary CB at 0.20% level significantly decreased the relative abundance of phylum Actinobacteria, class unidentified Actinobacteria, orders Bacillales, Lactobacillales and unidentified Gammaproteobacteria, family Burkholderiaceae, genus *Alcaligenes* and specie *Alcaligenes faecalis* (*P* < 0.05) (**Figures 4B, E**). Dietary CB at 0.40% level significantly increased the relative abundance of genera *Ralstonia* and *Nautella* and specie *Nautella italica* (*P* < 0.05) (**Figures 4C, F**).

## Discussion

There has been increasing evidence suggesting the maturation of intestinal tract, immune system and gut microbiota in the early life is significant for growth and development of marine fish larvae (34, 35). Early infancy is considered as an ideal window for gut microbial colonization (36, 37). Therefore, early intervention using probiotics during the critical period may be a promising method to enhance growth of fish larvae. In the present study, appropriate CB supplementation could enhance growth performance of large yellow croaker larvae, which were consistent with some previous findings in *Miichthys miiuy* (38), *Penaeus monodon* (39) and *Litopenaeus vannamei* (40).

The normal development of intestinal tract during the early life has a considerable impact on later health outcomes (1). The maturation process of gut tract can be enhanced, stopped or delayed depending on diet composition (19). In this study, CB supplementation could enlarge the villus height, enterocyte height, and muscular thickness of larval gut tract, which was consisted with some previous studies on tilapia (15), pacific white shrimp (40) and weaned piglets (41). Furthermore, the expression of *zo-2* and *odc* was higher in larvae fed diets with CB than the control group. *Odc* plays important roles in the proliferation, migration, and differentiation of cells (42); *zo-2* participates in tight junction structural integrity by binding to the actin cytoskeleton (43). These results suggested that CB could promote intestinal development of larvae by accelerating the proliferation of intestinal epithelial and maintaining the integrity of the intestinal barrier.

Moreover, digestive enzyme activities could be used as an indicator of development rate, food acceptance and digestive capacity (1). Previous research has indicated that CB could improve digestive function through producing a range of supplemental digestive enzymes (10, 39). In this study, 0.20% CB supplementation not only increased lipase in PS and IS of 37 DAH larvae, but also increased lipase in IS of 47 DAH larvae, which were consistent with the previous studies in broiler chickens (44), piglets (41), *Macrobrachium rosenbergii* (17) and *Litopenaeus vannamei* (40). Furthermore, the onset of BBM enzymes in intestine was considered as crucial in the maturation process of the digestive function (45). The present study found that 0.20% CB supplementation could improve activities of LAP in BBM, which demonstrated that appropriate dietary CB could promote the transition of larval digestive functions to adult mode (1).

The immune system during the early life is immature, which has been considered as a major restriction to healthy growth of fish larvae (46, 47). Probiotics can act as non-specific immune factors and have a positive effect on host immune system (35, 48) CB supplementation significantly affected nearly all immune enzyme activities (ACP, AKP, CAT and LZM), indicating CB supplementation could improve immune status of larvae. Intriguingly, no significant differences in the survival rate and mRNA expression of inflammatory factors were observed among dietary treatments. Zhao et al. (49) found CB could decrease cytokine levels (*ifn-γ*, *il-1β*, *il-8*, and *tnf-α*) in intestinal tissues of chickens with *Salmonella* infection, but no significant differences in cytokine levels were observed between CB treatment group and normal diet group (49). Above all, combining the results of the present study and current research, we speculated dietary CB could effectively decrease inflammatory factor level to protect host from pathogens.

Gut microbiota plays a crucial role in health and well-being of marine fish starting from hatch (4, 8). The current study indicated that modulation of intestine microbiota in large yellow croaker towards potentially more beneficial microbial community could be achieved through CB supplementation. The present study showed CB had a strong effect on the overall structure of intestinal microbiota in larvae. Similarly, compared with the control group, observed species number, PD whole tree index and ACE index were lower in the gut of larvae fed diets with CB supplementation. These results indicated that CB supplementation could regulate the structure of gut microbiota and decrease intestinal microbial diversity and richness, representing the positive effect of CB on gut microbiota development during the early life of larvae (35, 50).

Moreover, early life intervention using CB supplementation improved the composition of gut microbiota. CB supplementation at 0.10 and 0.20% levels significantly decreased the relative abundance of phylum Actinobacteria, indicating 0.10 and 0.20% CB may have a more profound effect on the intestinal community of larvae than 0.40% CB. Intriguingly, the present study found CB supplementation could increase the relative abundance of CB, which indicated larval intestine could be colonized by CB. Previous research has indicated that CB decreased the number of pathogens in the host gut tract by creating an oxygen-free environment, producing prebiotics and generating butyric acid (10). The present study found that CB significantly down-regulated the relative abundance of genus *Acinetobacter*, and CB at 0.20% significantly decreased the relative abundance of genus *Alcaligenes* and specie *Alcaligenes faecalis*. *Acinetobacter* was associated with bacteremia, pulmonary infections, meningitis, diarrhea, and nosocomial infections (51). And *Alcaligenes faecalis* was an opportunistic pathogen, which could be resistant to common antibiotics and closely related with nosocomial infections (52). These results revealed that CB could colonize in the fish larval gut tract, and play an inhibitory effect on opportunistic pathogens.

## Conclusion

This study demonstrated that early life intervention using dietary CB could improve growth performance of large yellow croaker larvae probably through promoting intestinal development, improving immune enzyme activities, and modulating gut microbiota. The optimal CB (containing cells at 5 × 10^9^ CFU g^−1^) supplementation dosage is 0.10–0.20%. Probiotic treatment in early life intervention may be a promising method to promote the maturation of the intestinal tract, immune system, and gut microbiota.

## Data Availability Statement

The datasets presented in this study can be found in online repositories. The names of the repository/repositories and accession number(s) can be found below: NCBI BioProject, accession no: PRJNA686247.

## Ethics Statement

The animal study was reviewed and approved by the Committee on the Ethics of Animal Experiments of Ocean University of China.

## Author Contributions

KM, QA, and ZY designed the research. ZY, QL, and YH conducted the research. SG, WH, CY, and ZY analyzed the data. ZY wrote the manuscript. QA and YG provided language help. All authors contributed to the article and approved the submitted version.

## Funding

This research was supported by the Agriculture Research System of China (Grant NO. CARS-47-11), the National Science Fund for Distinguished Young Scholars of China (Grant NO. 31525024), and the Key Program of National Natural Science Foundation of China (Grant NO. 31830103).

## Conflict of Interest

The authors declare that the research was conducted in the absence of any commercial or financial relationships that could be construed as a potential conflict of interest.

## Supplementary Material

Supplementary Figure 1 Effects of early life intervention using Clostridium butyricum on morphology of the intestine of large yellow croaker larvae (n = 3/group) (MT, muscular thickness; VH, villus height; EH, Enterocyte height). **(A)** Control group (0.00%); **(B)** CB1 group (0.10%); **(C)** CB2 group (0.20%); **(D)** CB3 group (0.40%); (HE staining; Scale bar = 100 mm).

Supplementary Figure 2 Effects of early life intervention using Clostridium butyricum on il-1b, il-6, il-8, ifng and cox-2 mRNA expression in whole intestine of large yellow croaker larvae. Values are means (n = 3), with their standard errors represented by vertical bars. Bars bearing the same letters were not significantly different (P >0.05, Tukey’s test).

Supplementary Figure 3 Rarefaction curve of gut microbiota of large yellow croaker larvae (n = 3/group).The rarefied curves for OTU number tended to approach the saturation plateau.

## References

[1] Zambonino Infante JL Cahu CL. Ontogeny of the gastrointestinal tract of marine fish larvae. Comp Biochem Physiol Part C: Toxicol Pharmacol (2001) 130:477–87. 10.1016/s1532-0456(01)00274-5 11738635

[2] Sánchez-Hernández J Nunn A Adams C Amundsen P-A. Causes and consequences of ontogenetic dietary shifts: a global synthesis using fish models. Biol Rev (2019) 94:539–54. 10.1111/brv.12468 30251433

[3] Comabella Y Hernández Franyutti A Hurtado A Canabal J García-Galano T. Ontogenetic development of the digestive tract in Cuban gar (*Atractosteus tristoechus*) larvae. Rev Fish Biol Fisheries (2012) 23:245–60. 10.1007/s11160-012-9289-z

[4] Stephens WZ Burns AR Stagaman K Wong S Rawls JF Guillemin K. The composition of the zebrafish intestinal microbial community varies across development. ISME J (2016) 10:644–54. 10.1038/ismej.2015.140 PMC4817687 26339860

[5] Chung H Pamp SünjeJ Hill JA Surana NK Edelman SM Troy EB. Gut Immune Maturation Depends on Colonization with a Host-Specific Microbiota. Cell (2012) 149:1578–93. 10.1016/j.cell.2012.04.037 PMC3442780 22726443

[6] Arrieta M-C Stiemsma L Amenyogbe N Brown E Finlay B. The intestinal microbiome in early life: health and disease. Front Immunol (2014) 5:427. 10.3389/fimmu.2014.00427 25250028 PMC4155789

[7] Dogra SK Sakwinska O Soh S-E Ngom-Bru C Brück W Berger B. Dynamics of infant gut microbiota are influenced by delivery mode and gestational duration and are associated with subsequent adiposity. mBio (2015) 6:e02419–02414. 10.1128/mBio.02419-14 PMC4323417 25650398

[8] Nguyen T Chung H-J Kim H-J Hong S-T. Establishment of an ideal gut microbiota to boost healthy growth of neonates. Crit Rev Microbiol (2019) 45:1–12. 10.1080/1040841X.2018.1561643 30773108

[9] Roggero P Liotto N Pozzi C Braga D Troisi J Menis C. Analysis of immune, microbiota and metabolome maturation in infants in a clinical trial of *Lactobacillus paracasei* CBA L74-fermented formula. Nat Commun (2020) 11:2703. 10.1038/s41467-020-16582-1 32483147 PMC7264213

[10] Duan Y Wang Y Dong H Ding X Liu Q Li H. Changes in the Intestine Microbial, Digestive, and Immune-Related Genes of *Litopenaeus vannamei* in Response to Dietary Probiotic *Clostridium butyricum* Supplementation. Front Microbiol (2018) 9:2191. 10.3389/fmicb.2018.02191 30283419 PMC6156435

[11] Tran NT Li Z Ma H Zhang Y Zheng H Gong Y. *Clostridium butyricum*: a promising probiotic confers positive health benefits in aquatic animals. Rev Aquaculture (2020) 12:2573–89. 10.1111/raq.12459

[12] Ling Z Liu X Cheng Y Luo Y Yuan L Li L. *Clostridium butyricum* Combined with *Bifidobacterium infantis* Probiotic Mixture Restores Fecal Microbiota and Attenuates Systemic Inflammation in Mice with Antibiotic-Associated Diarrhea. BioMed Res Int (2015) 2015:582048. 10.1155/2015/582048 25802855 PMC4352745

[13] Wang K Cao G Zhang H Li Q Yang C. Effects of *Clostridium butyricum* and *Enterococcus faecalis on* growth performance, immune function, intestinal morphology, volatile fatty acids, and intestinal flora in a piglet model. Food Funct (2019) 10:7844–54. 10.1039/c9fo01650c 31793606

[14] Xiang Q Wu X Pan Y Wang L Cui C Guo Y. Early-Life Intervention Using Fecal Microbiota Combined with Probiotics Promotes Gut Microbiota Maturation, Regulates Immune System Development, and Alleviates Weaning Stress in Piglets. Int J Mol Sci (2020) 21:503. 10.3390/ijms21020503 PMC7014131 31941102

[15] Poolsawat L Li X He M Ji D Leng X. *Clostridium butyricum* as probiotic for promoting growth performance, feed utilization, gut health and microbiota community of tilapia (*Oreochromis niloticus* × *O. aureus*). Aquaculture Nutr (2019) 26:657–70. 10.1111/anu.13025

[16] Li H Tian X Zhao K Jiang W Dong S. Effect of *Clostridium butyricum in* different forms on growth performance, disease resistance, expression of genes involved in immune responses and mTOR signaling pathway of *Litopenaeus vannamai* . Fish Shellfish Immunol (2019) 87:13–21. 10.1016/j.fsi.2018.12.069 30599253

[17] Sumon MS Ahmmed F Khushi SS Ahmmed MK Rouf MA Chisty MAH. Growth performance, digestive enzyme activity and immune response of *Macrobrachium rosenbergii* fed with probiotic *Clostridium butyricum* incorporated diets. J King Saud Univ - Sci (2018) 30:21–8. 10.1016/j.jksus.2016.11.003

[18] Yang B Zhou Y Wu M Li X Ai Q. ω-6 Polyunsaturated fatty acids (linoleic acid) activate both autophagy and antioxidation in a synergistic feedback loop via TOR-dependent and TOR-independent signaling pathways. Cell Death Dis (2020) 11:607. 10.1038/s41419-020-02750-0 32732901 PMC7393504

[19] Mai K Yu H Ma H Duan Q Gisbert E Infante JLZ. A histological study on the development of the digestive system of *Pseudosciaena crocea* larvae and juveniles. J Fish Biol (2005) 67:1094–106. 10.1111/j.0022-1112.2005.00812.x

[20] Liu Y Miao Y Xu N Ding T Cui K Chen Q. Effects of dietary *Astragalus* polysaccharides (APS) on survival, growth performance, activities of digestive enzyme, antioxidant responses and intestinal development of large yellow croaker (*Larimichthys crocea*) larvae. Aquaculture (2020) 517:734752. 10.1016/j.aquaculture.2019.734752

[21] Crane RK Boge G Rigal A. Isolation of brush border membranes in vesicular form from the intestinal spiral valve of the small dogfish (*Scyliorhinus canicula*). Biochim Biophys Acta (BBA) - Biomembranes (1979) 554:264–7. 10.1016/0005-2736(79)90024-5 454603

[22] Maroux S Louvard D Baratti J. The aminopeptidase from hog intestinal brush border. Biochim Biophys Acta (BBA) - Enzymol (1973) 321:282–95. 10.1016/0005-2744(73)90083-1 4750768

[23] Holm H Hanssen LE Krogdahl Å. Florholmen J. High and Low Inhibitor Soybean Meals Affect Human Duodenal Proteinase Activity Differently: In Vivo Comparison with Bovine Serum Albumin. J Nutr (1988) 118:515–20. 10.1093/jn/118.4.515 2451718

[24] Zuo R Ai Q Mai K Xu W Wang J Xu H. Effects of dietary n-3 highly unsaturated fatty acids on growth, nonspecific immunity, expression of some immune related genes and disease resistance of large yellow croaker (*Larmichthys crocea*) following natural infestation of parasites (*Cryptocaryon irritans*). Fish Shellfish Immunol (2012) 32:249–58. 10.1016/j.fsi.2011.11.005 22126857

[25] Li Q Cui K Wu M Xu D Mai K Ai Q. Polyunsaturated Fatty Acids Influence LPS-Induced Inflammation of Fish Macrophages Through Differential Modulation of Pathogen Recognition and p38 MAPK/NF-κB Signaling. Front Immunol (2020) 11:559332:559332. 10.3389/fimmu.2020.559332 33123132 PMC7572853

[26] Zhang W Zhang M Cheng A Hao E Huang X Chen X. Immunomodulatory and antioxidant effects of *Astragalus polysaccharide* liposome in large yellow croaker (*Larimichthys crocea*). Fish Shellfish Immunol (2020) 100:126–36. 10.1016/j.fsi.2020.03.004 32142872

[27] Livak KJ Schmittgen TD. Analysis of relative gene expression data using real-time quantitative PCR and the 2(-Delta Delta C(T)) Method. Methods (2001) 25:402–8. 10.1006/meth.2001.1262 11846609

[28] Ge H Wang Q Chen H Liu G Pan Y Chen J. Effects of antimicrobial peptide APSH-07 on the growth performance, anti-oxidation responses, stress resistance and intestine microbiota in large yellow croaker *Larimichthys crocea* . Aquaculture Nutr (2019) 26:715–26. 10.1111/anu.13031

[29] Magoc T Salzberg S. FLASH: fast length adjustment of short reads to improve genome assemblies. Bioinformatics (2011) 27:2957–63. 10.1093/bioinformatics/btr507 PMC3198573 21903629

[30] Caporaso JG Kuczynski J Stombaugh J Bittinger K Bushman FD Costello EK. QIIME allows analysis of high-throughput community sequencing data. Nat Methods (2010) 7:335–6. 10.1038/nmeth.f.303 PMC3156573 20383131

[31] Edgar RC. UPARSE: highly accurate OTU sequences from microbial amplicon reads. Nat Methods (2013) 10:996–8. 10.1038/nmeth.2604 23955772

[32] Desantis TZ Hugenholtz P Larsen N Rojas M Brodie EL Keller K. Greengenes, a chimera-checked 16S rRNA gene database and workbench compatible with ARB. Appl Environ Microbiol (2006) 72:5069–72. 10.1128/aem.03006-05 PMC1489311 16820507

[33] Segata N Izard J Waldron L Gevers D Miropolsky L Garrett WS. Metagenomic biomarker discovery and explanation. Genome Biol (2011) 12:R60. 10.1186/gb-2011-12-6-r60 21702898 PMC3218848

[34] Bentzon-Tilia M Sonnenschein EC Gram L. Monitoring and managing microbes in aquaculture - Towards a sustainable industry. Microbial Biotechnol (2016) 9:576–84. 10.1111/1751-7915.12392 PMC4993175 27452663

[35] Borges N Keller-Costa T Sanches-Fernandes GMM Louvado A Gomes NCM Costa R. Bacteriome Structure, Function, and Probiotics in Fish Larviculture: The Good, the Bad, and the Gaps. Annu Rev Anim Biosci (2021) 9:423–52. 10.1146/annurev-animal-062920-113114 33256435

[36] Gensollen T Iyer S Kasper D Blumberg R. How colonization by microbiota in early life shapes the immune system. Science (2016) 352:539–44. 10.1126/science.aad9378 PMC5050524 27126036

[37] Martínez I Maldonado-Gómez M Gomes-Neto J Kittana H Ding H Schmaltz R. Experimental evaluation of the importance of colonization history in early-life gut microbiota assembly. eLife (2018) 7:e36521. 10.7554/eLife.36521 30226190 PMC6143339

[38] Song ZF Wu TX Cai LS Zhang LJ Zheng XD. Effects of dietary supplementation with *Clostridium butyricum* on the growth performance and humoral immune response in *Miichthys miiuy* . J Zhejiang Univ Sci B (2006) 7:596–602. 10.1631/jzus.2006.B0596 16773736 PMC1500882

[39] Duan Y Zhang J Huang J Jiang S. Effects of Dietary *Clostridium butyricum* on the Growth, Digestive Enzyme Activity, Antioxidant Capacity, and Resistance to Nitrite Stress of *Penaeus monodon* . Probiotics Antimicrob Proteins (2019) 11:938–45. 10.1007/s12602-018-9421-z 29858778

[40] Duan Y Zhang Y Dong H Wang Y Zheng X Zhang J. Effect of dietary *Clostridium butyricum* on growth, intestine health status and resistance to ammonia stress in Pacific white shrimp *Litopenaeus vannamei* . Fish Shellfish Immunol (2017) 65:25–33. 10.1016/j.fsi.2017.03.048 28359948

[41] Hu X Lin B Luo M Zheng X Zhang H. The isolation, identification, physiological property of pig-isolate *Clostridium butyricum* LY33 using lactic acid and its effects on intestinal function of weaned piglets. Ital J Anim Sci (2019) 18:910–21. 10.1080/1828051x.2019.1603089

[42] Singh K Coburn L Asim M Barry D Allaman M Shi C. Ornithine Decarboxylase in Macrophages Exacerbates Colitis and Promotes Colitis-Associated Colon Carcinogenesis by Impairing M1 Immune Responses. Cancer Res (2018) 78:4303–15. 10.1158/0008-5472.CAN-18-0116 PMC6072585 29853605

[43] Ou W Hu H Yang P Dai J Ai Q Zhang W. Dietary daidzein improved intestinal health of juvenile turbot in terms of intestinal mucosal barrier function and intestinal microbiota. Fish Shellfish Immunol (2019) 94:132–41. 10.1016/j.fsi.2019.08.059 31461659

[44] Zhang L Zhang L Zhan X Zeng X Zhou L Cao G. Effects of dietary supplementation of probiotic, *Clostridium butyricum*, on growth performance, immune response, intestinal barrier function, and digestive enzyme activity in broiler chickens challenged with *Escherichia coli* K88. J Anim Sci Biotechnol (2016) 7:3. 10.1186/s40104-016-0061-4 26819705 PMC4728939

[45] Ma H Cahu C Zambonino J Yu H Duan Q Le Gall MM. Activities of selected digestive enzymes during larval development of large yellow croaker (*Pseudosciaena crocea*). Aquaculture (2005) 245:239–48. 10.1016/j.aquaculture.2004.11.032

[46] Georgountzou A Papadopoulos NG. Postnatal innate immune development: From birth to adulthood. Front Immunol (2017) 8:957. 10.3389/fimmu.2017.00957 28848557 PMC5554489

[47] López Nadal A Ikeda-Ohtsubo W Sipkema D Peggs D Mcgurk C Forlenza M. Feed, Microbiota, and Gut Immunity: Using the Zebrafish Model to Understand Fish Health. Front Immunol (2020) 11:114. 10.3389/fimmu.2020.00114 32117265 PMC7014991

[48] Pérez-Sánchez T Ruiz-Zarzuela I De Blas I Balcázar JL. Probiotics in aquaculture: a current assessment. Rev Aquaculture (2014) 6:133–46. 10.1111/raq.12033

[49] Zhao X Yang J Ju Z Wu J Wang L Lin H. *Clostridium butyricum* Ameliorates *Salmonella Enteritis* Induced Inflammation by Enhancing and Improving Immunity of the Intestinal Epithelial Barrier at the Intestinal Mucosal Level. Front Microbiol (2020) 11:299. 10.3389/fmicb.2020.00299 32180765 PMC7059641

[50] Geraylou Z Souffreau C Rurangwa E De Meester L Courtin CM Delcour JA. Effects of dietary arabinoxylan-oligosaccharides (AXOS) and endogenous probiotics on the growth performance, non-specific immunity and gut microbiota of juvenile Siberian sturgeon (*Acipenser baerii*). Fish Shellfish Immunol (2013) 35:766–75. 10.1016/j.fsi.2013.06.014 23811408

[51] Hamuel JD Ndakidemi P Human I Benade S. The Ecology, Biology and Pathogenesis of *Acinetobacter* spp.: An Overview. Microbes Eenviron (2011) 26:101–12. 10.1264/jsme2.ME10179 21502736

[52] Ethica SN Semiarti E Widada J Oedjijono O Joko Raharjo T. Characterization of *moaC* and a nontarget gene fragments of food-borne pathogen *Alcaligenes* sp. JG3 using degenerate colony and arbitrary PCRs. J Food Saf (2017) 37:e12345. 10.1111/jfs.12345

