# Supplementary material for: Early Life Intervention Using Probiotic Clostridium butyricum Improves Intestinal Development, Immune Response, and Gut Microbiota in Large Yellow Croaker (Larimichthys crocea) Larvae
Source: Front Immunol. 2021 Mar 8;12:640767. doi: 10.3389/fimmu.2021.640767 (PMC7982665; doi:10.3389/fimmu.2021.640767)
Supplement: Supplementary file 4 [file Table_1.docx]

Supplementary Table 1 Formulation and proximate analysis of the experimental diets (% dry matter)

| Ingredient / % dry diet | Experimental diets (%) | | | |
| --- | --- | --- | --- | --- |
|  | Control | CB1 | CB2 | CB3 |
|  | 0.00% | 0.10% | 0.20% | 0.40% |
| White fish meal ^a^ | 36.00 | 36.00 | 36.00 | 36.00 |
| Krill meal ^b^ | 25.00 | 25.00 | 25.00 | 25.00 |
| Squid meal ^c^ | 8.50 | 8.50 | 8.50 | 8.50 |
| Microcrystalline Cellulose | 0.40 | 0.30 | 0.20 | 0.00 |
| Yeast extract ^a^ | 3.00 | 3.00 | 3.00 | 3.00 |
| Sodium alginate | 2.00 | 2.00 | 2.00 | 2.00 |
| α-starch | 7.60 | 7.60 | 7.60 | 7.60 |
| Vitamin premix ^d^ | 1.50 | 1.50 | 1.50 | 1.50 |
| Mineral premix ^e^ | 1.00 | 1.00 | 1.00 | 1.00 |
| Ascorbyl polyphosphate | 0.20 | 0.20 | 0.20 | 0.20 |
| Attractant mixture ^f^ | 2.00 | 2.00 | 2.00 | 2.00 |
| Mould inhibitor | 0.05 | 0.05 | 0.05 | 0.05 |
| Antioxidant | 0.05 | 0.05 | 0.05 | 0.05 |
| Choline choride | 0.20 | 0.20 | 0.20 | 0.20 |
| Fish oil | 6.50 | 6.50 | 6.50 | 6.50 |
| Soybean Lecithin | 6.00 | 6.00 | 6.00 | 6.00 |
| *Clostridium butyricum* ^g^ | 0.00 | 0.10 | 0.20 | 0.40 |
| Analyzed nutrients composition (dry matter basis %) | | | | |
| Ash | 14.24 | 14.71 | 14.53 | 14.66 |
| Crude fat | 19.66 | 19.29 | 19.46 | 19.12 |
| Crude protein | 51.57 | 51.94 | 51.83 | 51.94 |

^a^ Commercially available from Guangdong VTR Bio-Tech Co., Ltd. (Zhuhai, China); elementary composition (dry matter): White fish meal, crude protein, 71.73%, crude lipid, 4.76%.

^b^ Commercially available from Qingdao Bio-ways Ingredients Biotechnology Co., Ltd. (Qingdao, China); elementary composition (dry matter): Krill meal, crude protein, 64.86%, crude lipid, 8.0%.

^c^ Commercially available from Haixingyuan Feed Co., Ltd.Co., Ltd. (Hebei, China); elementary composition (dry matter): Squid meal, crude protein, 81.81%, crude lipid, 5.16%.

^d^ Composition of vitamin premix (IU or g ^kg−1^): vitamin A palmitate, 3000000 IU; vitamin D_3_ 1200000 IU; DL-α-vitamin E 40.0 g kg^-1^; menadione, 8.0 g kg^-1^; thiamine-HCl, 5.0 g kg^-1^; riboflavin, 5.0 g kg^-1^; D-calcium pantothenate, 16.0 mg kg^-1^; pyridoxine-HCl, 4.0 mg kg^-1^; inositol, 200.0 mg kg^-1^; biotin, 8.0 mg kg^-1^; folic acid, 1.5 mg kg^-1^; 4-aminobenzoic acid, 5.0 mg kg^-1^; niacin, 20.0 mg kg^-1^; vitamin B_12_, 0.01 mg kg^-1^; L-ascorgyl-2-monophosphate-Na (3%), 2000.0 mg kg^-1^.

^e^ Composition of mineral premix (g kg^−1^ premix): Ca(H_2_PO_4_)·H_2_O, 675.0; C_O_SO_4_·H_2_O, 0.15; CuSO_4_·H_2_O, 5.0; FeSO_4_·7H_2_O, 50.0; KCl, 0.1; MnSO_4_·2H_2_O, 101.7; MnSO_4_·2H_2_O, 18.0; NaCl, 80.0; NaSeO_3_·H_2_O, 0.05; ZnSO_4_·7H_2_O, 20.0.

^f^ Commercially available from Qingdao Qihao biotechnology co., Ltd. (Qingdao, China).

^g^ Commercially available from Vland Biotech Co., Ltd., China, containing cells at 5×10^9^ CFU g^−1^.
