# Supplementary material for: Early Life Intervention Using Probiotic Clostridium butyricum Improves Intestinal Development, Immune Response, and Gut Microbiota in Large Yellow Croaker (Larimichthys crocea) Larvae
Source: Front Immunol. 2021 Mar 8;12:640767. doi: 10.3389/fimmu.2021.640767 (PMC7982665; doi:10.3389/fimmu.2021.640767)
Supplement: Supplementary file 5 [file Table_2.docx]

Supplementary Table 2 Primers used for quantitative PCR.

| Gene | Forward (5'-3') | Reverse (3'-5') | Reference |
| --- | --- | --- | --- |
| *zo-1* ^2^ | TGTCAAGTCCCGCAAAAATG | CAACTTGCCCTTTGACCTCT | XM019260744 |
| *zo-2* ^2^ | ACCCGACCTGTTTGTTATTG | ATGCCGTGCTTGCTGTC | (Liu et al., 2020) |
| *occludin* | AGGCTACGGCAACAGTTATG | GTGGGTCCACAAAGCAGTAA | XM010740442 |
| *pcna* ^2^ | AGTTTGCCCGTATCTGCC | CTCTTTGTCTACATTGCTGGTCT | (Liu et al., 2020) |
| *odc* ^2^ | GAGCCAGGTCGCTTCTATG | CCGTGGTCCCTTCGTCT | (Liu et al., 2020) |
| *il-1β* ^2^ | CATAGGGATGGGGACAACGA | AGGGGACGGACACAAGGGTA | (Li et al., 2020) |
| *il-6* ^2^ | CGACACACCCACTATTTACAAC | TCCCATTTTCTGAACTGCCTCT | (Li et al., 2020) |
| *il-8* ^2^ | CTATCGTGGCACTCCTGGTT | GCAGGAATCACCTCCACTTGT | (Zhang et al., 2020) |
| *ifnγ* ^2^ | TCAGACCTCCGCACCATCA | GCAACCATTGTAACGCCACTTA | (Li et al., 2020) |
| *cox-2* ^2^ | CTGGAAAGGCAACACAAGC | CGGTGAGAGTCAGGGACAT | (Li et al., 2020) |
| *β-actin* | GACCTGACAGACTACCTCATG | AGTTGAAGGTGGTCTCGTGGA | (Liu et al., 2020) |

^2^ *zo-1*: tight zonula occludens-1; *zo-2*: tight zonula occludens-2; *pcna*: proliferating cell nuclear antigen; *odc*: ornithine decarboxylase; *il-1β*: interleukin-1β; *il-6*: interleukin-6; *il-8*: interleukin-8; *ifnγ*: interferon γ; *cox-2*: cyclooxygenase-2.
